# Image Phenotyping of Spring Barley (*Hordeum vulgare* L.) RIL Population Under Drought: Selection of Traits and Biological Interpretation

**DOI:** 10.3389/fpls.2020.00743

**Published:** 2020-06-09

**Authors:** Krzysztof Mikołajczak, Piotr Ogrodowicz, Hanna Ćwiek-Kupczyńska, Kathleen Weigelt-Fischer, Srinivasa Reddy Mothukuri, Astrid Junker, Thomas Altmann, Karolina Krystkowiak, Tadeusz Adamski, Maria Surma, Anetta Kuczyńska, Paweł Krajewski

**Affiliations:** ^1^Institute of Plant Genetics, Polish Academy of Sciences, Poznań, Poland; ^2^Leibniz Institute of Plant Genetics and Crop Plant Research (IPK), Gatersleben, Germany; ^3^Institute of Bioorganic Chemistry, Polish Academy of Sciences, Poznań, Poland

**Keywords:** automated high-throughput plant phenotyping, barley, data analysis methods, drought stress, dynamic traits

## Abstract

Image-based phenotyping is a non-invasive method that permits the dynamic evaluation of plant features during growth, which is especially important for understanding plant adaptation and temporal dynamics of responses to environmental cues such as water deficit or drought. The aim of the present study was to use high-throughput imaging in order to assess the variation and dynamics of growth and development during drought in a spring barley population and to investigate associations between traits measured in time and yield-related traits measured after harvesting. Plant material covered recombinant inbred line population derived from a cross between European and Syrian cultivars. After placing the plants on the platform (28th day after sowing), drought stress was applied for 2 weeks. Top and side cameras were used to capture images daily that covered the visible range of the light spectrum, fluorescence signals, and the near infrared spectrum. The image processing provided 376 traits that were subjected to analysis. After 32 days of image phenotyping, the plants were cultivated in the greenhouse under optimal watering conditions until ripening, when several architecture and yield-related traits were measured. The applied data analysis approach, based on the clustering of image-derived traits into groups according to time profiles of statistical and genetic parameters, permitted to select traits representative for inference from the experiment. In particular, drought effects for 27 traits related to convex hull geometry, texture, proportion of brown pixels and chlorophyll intensity were found to be highly correlated with drought effects for spike traits and thousand grain weight.

## Introduction

Barley (*Hordeum vulgare* ssp. *vulgare* L.) is one of the most important crops worldwide because of its multipurpose usage in human diet and as animal feed. Although it is known to adapt to a wide range of environments, in Europe new cultivars have been bred under favorable conditions, which led to the narrowing of genetic diversity in agronomical properties, including resistance to environmental stresses like shortage of water. The gene pool for tolerance to water scarcity in modern elite cultivars is very limited. Broadening of the genetic diversity provides the basis for plant improvement. Hence, success in breeding new varieties with improved tolerance to water shortage or heat can be achieved through the use of wild relatives, landraces or varieties growing in dry areas as donors of the resistance (Ceccarelli, 1994; Ceccarelli and Grando, 1999; Grando et al., 2001; Cattivelli et al., 2008).

Understanding adaptation of plants to their environment is a key issue that is addressed in many fields of study and may also contribute to breeding crop plants adapted to sub-optimal conditions. Water scarcity (also referred to as drought stress) is a condition associated with insufficient soil moisture available to provide satisfactory crop production. Development of drought tolerant cultivars becomes increasingly important in changing climate. Many different approaches have been used to study the nature of plant reactions to defined levels of drought stress, including physiological processes investigations (Buschmann et al., 2000; Sanchez et al., 2002; Jones et al., 2003) and phenotyping on conveyor systems in glasshouses with controlled irrigation systems (Tuberosa, 2012; Honsdorf et al., 2014).

Accurate quantification of traits observed in plants grown under conditions of limited watering is crucial for identifying loci of interest in the genome. While in construction of genetic maps high-throughput genotyping platforms are used routinely, plant phenotypes are still assessed mainly by conventional procedures, which are time-consuming, labor-intensive, low-throughput, and usually destructive. Studying the drought stress response is particularly challenging as its impact on plant performance is a dynamic process that occurs across space and time. Endpoint measurements are insufficient to asses and analyze dynamic responses. Thus, phenotyping has become the major operational bottleneck limiting the power of genetic analysis (Hartmann et al., 2011; Cabrera-Bosquet et al., 2012).

In recent years, automation, imaging, and software solutions have paved the way for many high-throughput phenotyping studies (Munns et al., 2010; Busemeyer et al., 2013; Chen et al., 2014; Honsdorf et al., 2014; Paulus et al., 2014). Regardless of the platform setting, the goal is to evaluate phenotypic properties of plants using automated processes in a non-invasive way. Automated systems have been successfully applied to investigate numerous components of plant growth, and may be used to help tackle basic research questions when combined with genetic strategies (Famoso et al., 2010; Muraya et al., 2017; Neumann et al., 2017).

The aim of the present study was to screen changes in growth, architecture and physiology traits measured by high-throughput imaging in spring barley RILs population under drought and control (well-watered) conditions, to identify traits relevant for the description of drought response and to assess an association between the traits measured during the time-course experiment and yield-related traits measured after harvest. The purpose was also to propose a method of selection of traits produced by image analysis in order to obtain a non-redundant feature set characterizing barley drought response. We assume that quantitative analyses of plant structure traits during development under water-limited environments will permit the identification of lines with enhanced resilience to water deficit.

## Materials and Methods

### Plant Material

Spring barley (*Hordeum vulgare* L.) recombinant inbred lines (RILs) population (hereafter referred to as MCam) derived from a cross between European and Syrian spring barley cultivars – Maresi and Cam/B1/CI08887//CI05761 – was used in our studies (Mikołajczak et al., 2016). Maresi is a German semi-dwarf cultivar with the pedigree Cebeco-6801/GB-1605//HA-46459-68, and Cam/B1/CI08887//CI05761 (hereafter referred to as Cam/B1/CI) is a Syrian breeding line adapted to dry environments. The Syrian genotype was supplied to Dr A. Górny (Institute of Plant Genetics PAS, Poznań) by Drs S. Grando and S. Ceccarelli from ICARDA in Aleppo, and European cultivar was obtained from the collection of IPG PAS Poznań. RILs were derived by the single-seed descent technique (Goulden, 1939) until F_8_ generation. 95 RILs and the parental genotypes Maresi and Cam/B1/CI were examined in the experiment.

### Plant Cultivation and Phenotyping

After 4 weeks of pre-cultivation, plants were transplanted onto a high-throughput platform. They grew under controlled greenhouse conditions and were phenotyped on a daily basis using the fully automated system consisting of conveyor belts, a weighing and watering station, and three imaging sensors. Each genotype was represented by 16 plants located in four carriers, each with four pots. Genotypes were randomized throughout the plant growth area. Two environmental conditions were simulated: control – optimal watering (for two carriers), and stress – limited watering (for another two carriers). The growth conditions in the greenhouse were set to 20°C during the day and 16°C at night with relative humidity >65%. The daylight period lasted 16 h starting at 6 AM. Using automated, target-weight based watering, control plants remained well-watered at a field capacity of 70%, and those in stress conditions were kept at 20% field capacity.

Drought stress was applied from the fifth day after placing the plants on the platform (35th day after sowing, DAS 35) until day 18th (DAS 48). After the stress period, plants were re-watered to optimal field capacity and kept well-watered again for another 2 weeks. Fertilization was carried out twice with Combo Hakaphos blau (200 cm^3^ absolute volume per plant). In order to avoid any position effects, the carriers were shuffled daily for one lane, every 3rd day within each lane for 11 positions. Imaging started 4 days after transplanting onto the platform (DAS 31) and was performed daily for 33 days until DAS 63. Observations were not done on DAS 56, but, for simplicity, the data were analyzed as a continuous series of 32 time points. One top and three side view images were taken covering the visible range of the light spectrum (VIS), static fluorescence signals (FLUOR), and the near infrared spectrum (NIR) (Junker et al., 2015). In this manner, 190,493 images were acquired for all genotypes and two treatments during the whole image phenotyping period. The image processing pipeline (Integrated Analysis Platform, IAP, Klukas et al., 2014) provided 376 traits that were subjected to analysis (Supplementary Table 1).

After imaging in the phenotyping platform, the plants were moved from the platform to a non-automated greenhouse, where they were cultivated for another 40 days under optimal watering conditions until ripening. After harvesting, 12 plant architecture- and yield-related traits were measured for each genotype on two plants grown in control conditions and two plants grown in drought (Table 1).

**TABLE 1:** Results of ANOVA and estimation of genetic parameters for traits observed after harvesting.

**Trait no.**	**Symbol**	**Trait**	**-log_10_(*P*-value) for testing of mean drought effect**	**Variance component for RILs in control conditions (std. err.)**	**Variance component for RILs under drought conditions (std. err.)**	**Genetic correlation between conditions**	**Phenotypic correlation between conditions**
1	PH	Plant height (cm)	39.39	0.76 (0.23)	0.22 (0.13)	0.71	0.31
2	NTP	Number of productive tillers	24.09	0 (–)	0 (–)	–	0.02
3	NTT	Total number of tillers	16.05	0 (–)	0 (–)	–	0.23
4	TGW	1000-grain weight (g)	13.60	0.78 (0.23)	1.85 (0.43)	0.57	0.40
5	LSm	Length of main spike (cm)	45.31	5.48 (1.07)	3.88 (0.78)	0.88	0.79
6	NSm	Number of spikelets per main spike	45.09	9.78 (1.83)	4.71 (0.93)	0.89	0.82
7	NGm	Number of grains per main spike	42.42	4.54 (0.90)	2.73 (0.58)	0.71	0.62
8	GWm	Grain weight per main spike (g)	43.48	7.40 (1.41)	2.71 (0.57)	0.65	0.58
9	LSl	Length of lateral spike (cm)	41.18	3.50 (0.71)	2.09 (0.46)	0.91	0.77
10	NSl	Number of spikelets per lateral spike	47.44	3.93 (0.79)	2.54 (0.54)	0.94	0.81
11	NGl	Number of grains per lateral spike	47.41	2.23 (0.49)	1.06 (0.28)	0.58	0.43
12	GWl	Grain weight per lateral spike (g)	43.22	3.42 (0.70)	0.70 (0.21)	0.47	0.34

*Variance components for RILs estimated in the mixed model with unstructured covariance matrix for GE interaction; 0 – negative estimates of variance components.*

### Statistical Analysis

Data (per-plant observations) obtained from the image analysis pipeline were first submitted to outlier removal by application of the Grubbs test (Grubbs, 1950). Then, data were averaged over plants within carriers giving as input for statistical analysis, for each trait, a table for 97 genotypes × 2 conditions × 2 biological replications (carriers) × 32 days. Pearson correlation coefficients between time courses of observations done in replicates (carriers of the same genotype) were computed and only the traits for which both the correlation between two carriers of non-stressed plants and the correlation between two carriers of stressed plants were both >0.2 were selected for further analysis. A linear mixed model with fixed effects of treatment and random effects of genotype × treatment interaction (with unstructured covariance matrix for treatment term) was fitted for data obtained on each day, with REML estimation of variance components, estimation of genetic correlation between conditions, and Wald test of mean drought effect. Relative drought effects (RDE) for genotypes were computed as 100^∗^(mean value under drought – mean value under control)/mean value under control. Grouping of image-based traits with respect to time profiles of computed parameters (drought effects, genetic correlations, correlations with after-harvest traits) was performed by searching for the optimum clustering into 1, 2, …, 10 groups using the minimum within-group sum of squares (WSS) criterion and selecting the proper number of groups by inspection of the scree plot of WSS values.

Phenotypic data obtained after harvesting were analyzed by a mixed linear model analogous to the one used for image-derived data. Pearson correlation coefficients were computed between RDE for each after-harvest trait (computed as above) and each image trait on each day (using the sets of RDE for all genotypes). Prediction of RDE for after-harvest traits by RDE for image-based traits was done by fitting a partial least squares (PLS) regression model with two-groups cross-validation.

Data processing until obtaining of mean values over carriers was done in R 3.3.1 (R Core Team, 2018). Subsequent statistical analyses and visualizations (violin plots, principal component biplots, correlation heatmaps) were performed in Genstat 17 (VSN International, 2013).

## Results

### Analysis of Post-harvest Traits

For all 12 traits observed after harvesting, the distributions of mean values for lines under drought was shifted to lower values (Supplementary Figure 1A), which resulted in significant mean drought effects (Table 1; *P* < 0.001). The variance components for RILs were the largest for traits describing main spikes (LSm, NSm, NGm, GWm) and larger under control than under stress conditions for all traits except TGW (Table 1; see also differences in range of distributions shown in Supplementary Figure 1A). The genetic correlation between conditions was the largest for length of spikes and for number of spikelets (LSm, LSl, NSm, NSl), and the smallest for GWl and TGW. Phenotypic correlation between conditions was very low for number of tillers (NTP, NTT); genetic correlation could not be estimated for these traits due to the presence of negative estimates of variance components. The biplot in Supplementary Figure 1B shows a smaller role of traits describing the numbers of tillers than of other traits in discriminating non-stressed and stressed plants.

Analysis of correlations between traits (Supplementary Figure 1C) revealed high positive correlations between both traits describing number of tillers (NTP, NTT) and between traits describing spike properties, with correlations larger in control conditions than under stress. Correlation coefficients revealed a weak association between plant height and other traits both in non-stress and stress conditions, although in drought, this association appeared to be slightly stronger. Total number of tillers in non-stress conditions was negatively correlated with spike traits, i.e., length, number of spikelets and grains in main and lateral spikes, as well as thousand kernel weight, but in drought stress, these correlations became weaker.

**FIGURE 1:**
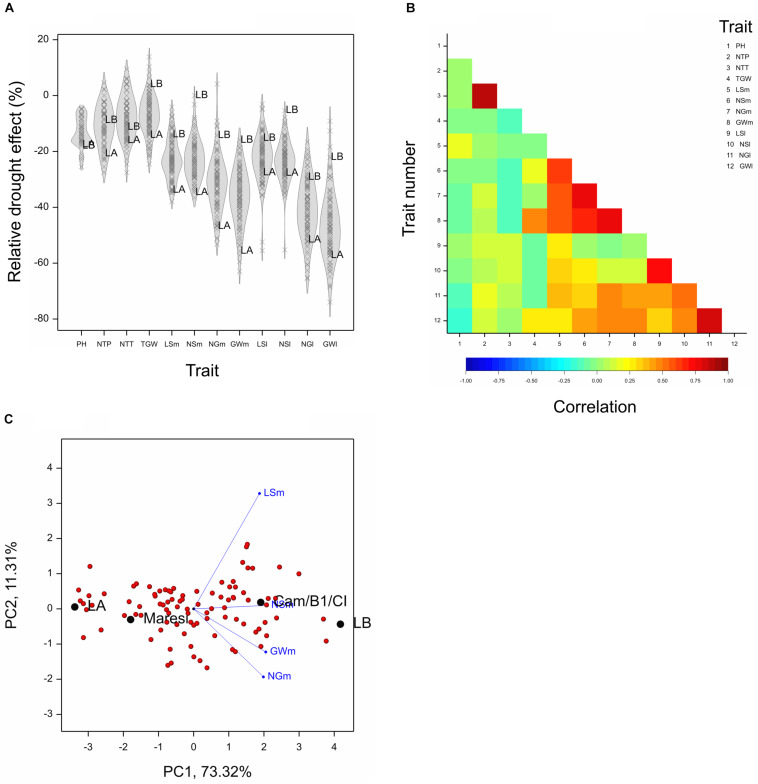
Results concerning relative drought effects for traits measured after harvesting (traits designated by abbreviations listed in Table 1). **(A)** Density plots for relative effects for traits. Marked lines LA, LB. **(B)** Correlations between relative effects for pairs of traits. Critical value of correlation at *P* < 0.01 is approximately ±0.235. **(C)** Biplot made on the basis of drought effects for four traits describing main spikes.

For the purpose of further analysis, relative drought effects (RDE) were computed for all lines and all after-harvesting traits; they were on average negative (Figure 1A). Strong correlation of effects was found for two traits describing numbers of tillers and for subsets of traits describing main and lateral spikes (Figure 1B). On the basis of traits with largest inter-line variation, i.e., four main spike traits (LSm, NSm, NGm, GWm), we identified two lines that were very distant in optimal conditions and quite similar in drought, therefore, contrasting with respect to reaction to drought: LA (MCam001) with large trait values under control conditions and a large relative loss under drought, and LB (MCam079) with low values under control and small relative loss under drought (Supplementary Figures 1A,B). These two lines are extreme in terms of the scores of the first principal component obtained for RDE of main spike traits (Figure 1C and Supplementary Table 2). Lines LA and LB were contrasting in a similar manner also with respect to traits concerning lateral spikes, TGW (note a positive drought effect for LB, Figure 1A) and, to a smaller extent, number of tillers. Line LB was somewhat taller than LA, but the drought effects for plant height were similar for both lines. Parental lines Maresi and Cam/B1/CI were not as extreme as LA and LB, which indicates existence of transgression effects with respect to reaction to drought.

### Image-Based Phenotyping of the Dynamics in Drought Response

Out of 376 characteristics provided by the image analysis pipeline, seven contained no observations, and 83 provided data that did not satisfy the criterion requesting the correlation between carriers to be bigger than 0.2 (Supplementary Table 1). The classification of all image-extracted traits with respect to their type and origin is shown in Table 2. One can note that a rather small proportion of traits derived from near infrared imaging (41.18%) was accepted for further analysis.

**TABLE 2:** Classification of traits according to type and acceptance for analysis on the basis of correlations between carriers.

**Classification**	**Number of traits**		**% accepted**	**Total number of traits**
	**Not accepted**	**Accepted**		
**Camera type**				
VIS	50	146	74.49	196
FLUOR	30	132	81.48	162
NIR	10	7	41.18	17
MULTI*	0	1	100.00	1
Total	90	286	76.06	376
**Category**				
Color	4	108	96.43	112
Texture	2	46	95.83	48
Geometric	84	156	65.00	240
Total	90	286	76.06	376
**Statistic**				
First order	90	238	72.56	328
St. dev.	0	24	100.00	24
Skewness	0	24	100.00	24
Total	90	286	76.06	376
**Color scale**				
None	86	180	67.67	266
RGB	2	28	93.33	30
HSV	2	42	95.45	44
LAB	0	36	100.00	36
Total	90	286	76.06	376
**Viewpoint**				
Side	42	143	77.30	185
Top	44	131	74.86	175
Combined	4	12	75.00	16
Total	90	286	76.06	376

*Trait no. 316 computed from VIS and NIR camera images.

**FIGURE 2:**
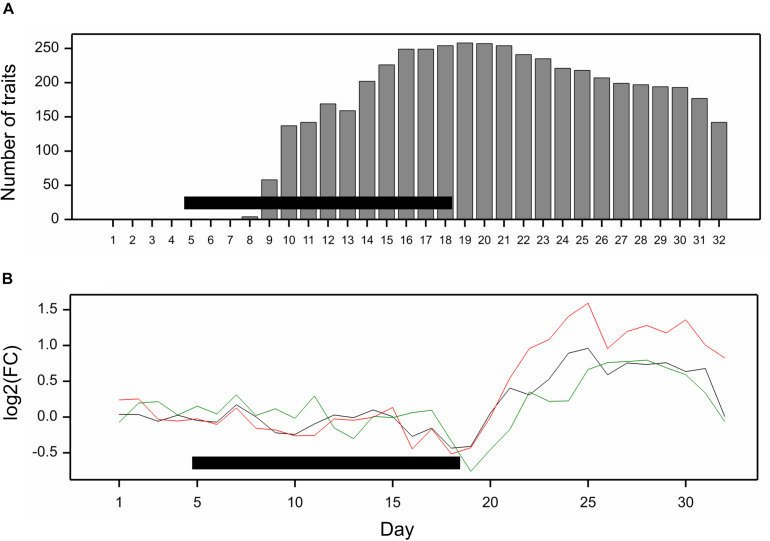
**(A)** Number of traits with significant mean drought effect on consecutive days of experiment; significance assessed by *F*-test at *p* < 0.05 with Bonferroni approximation. **(B)** Fold change of genetic variance under drought to genetic variance under optimal conditions on consecutive days. The lines show profiles of median log_2_FC for color (black), texture (red), and geometric (green) traits. Horizontal bars mark the drought treatment period.

**FIGURE 3:**
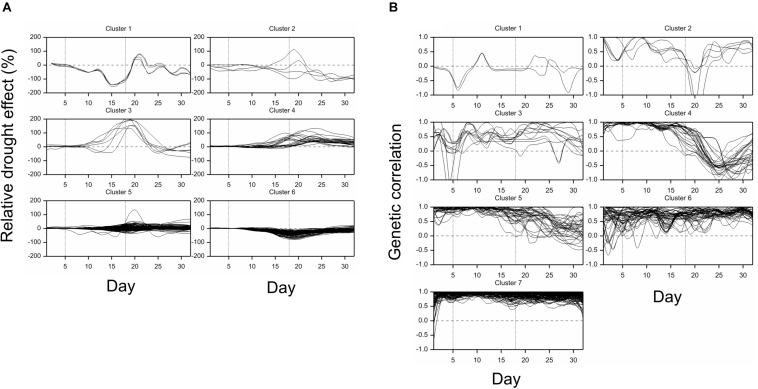
Time profiles of **(A)** mean drought effects (averaged over all genotypes), and **(B)** genetic correlation coefficients between optimal and drought conditions, for traits measured in time, clustered using minimum within-group sum of squares (WSS) criterion. Smooth lines drawn using splines with 16 d.f., vertical dashed lines mark start and end of the drought treatment period.

All traits but one (no. 21, “leaf length”) exhibited a significant mean drought effect at least at one time point (ANOVA *F*-test, *p* < 0.05, Bonferroni correction over 286 analyzed traits). First significant effects were observed on the fourth day of drought, and the number of traits with a significant effect grew until first day after drought (Figure 2A). Clustering of time profiles of average RDE revealed six groups of traits (Figure 3A; Supplementary Figure 2A) differing by the shape and effect size. The largest and consistent drought effects were observed in clusters 1 and 3. Cluster no. 1 contained four highly similar profiles of negative effects for traits measured in visible or fluorescent light describing projected plant area (traits 38, 44, 45, 277). Cluster no. 3 contained six profiles for traits describing proportion of yellow, red or brown pixels (traits 139, 142, 144, 324) and skewness of colors (traits 328, 334), measured in visible light by side and top cameras, with positive drought effect increasing from about 12 to 18 day, meaning increased intensity of these colors under drought (plants less green). Groups 4, 5, and 6 contained profiles for, respectively, 26, 116, and 130 traits, with positive (group 4; all but one were fluorescent traits) and negative (group 6; 90% were geometric traits) drought effects. In terms of drought effects progression, for most of the traits, the maximum effects were observed at the end of drought period, but there was a group of traits in cluster 4 with a large drought effect appearing about 5 days later (traits 124, 132, 166, 167, 284, 286, 297, 301, 302, 308, 309, 317, 344, 345). Clusters 2 and 4 were composed of traits that indicated a lack of recovery of plants by the end of the observation period.

The differences between genetic variance in drought and control, measured by the fold change in logarithmic scale (log_2_FC), were visible, especially after 18th day (end of drought period). The median values of FC (over traits) first slightly decreased, but then increased, especially for texture traits (Figure 2B), meaning bigger variance among lines subjected to drought for many image-derived features. Clustering of time profiles of genetic correlation between conditions (GC), the indicator of the genotype by environment (GE) interaction, revealed seven groups of traits (Figure 3B; Supplementary Figure 2B). A temporary decrease of GC after the end of the drought period could be seen in cluster 2 containing five traits (153, 160, 181, 287, 288; color uniformity, texture, skewness of saturation). In cluster 4 (28 traits; 46% texture traits, 75% top camera), a very strong decrease of correlation to values close to -1 (indicating a strong cross-over interaction) with a subsequent increase to values close to zero was observed. For traits in group 5 (34 traits; 67% color, 2 geometric traits: compactness 245, area/skeleton length 272), the correlation started decreasing at the end of drought period. For three traits in group 1 (43, 44, 45; projected plant area), the correlation was small on all days; for the rest of the traits, in groups 6 (80% geometric traits, 82% top camera) and 7 (64% geometric, 62% side camera), the correlations were close to 1 on all days, indicating a lack of GE interaction.

**FIGURE 4:**
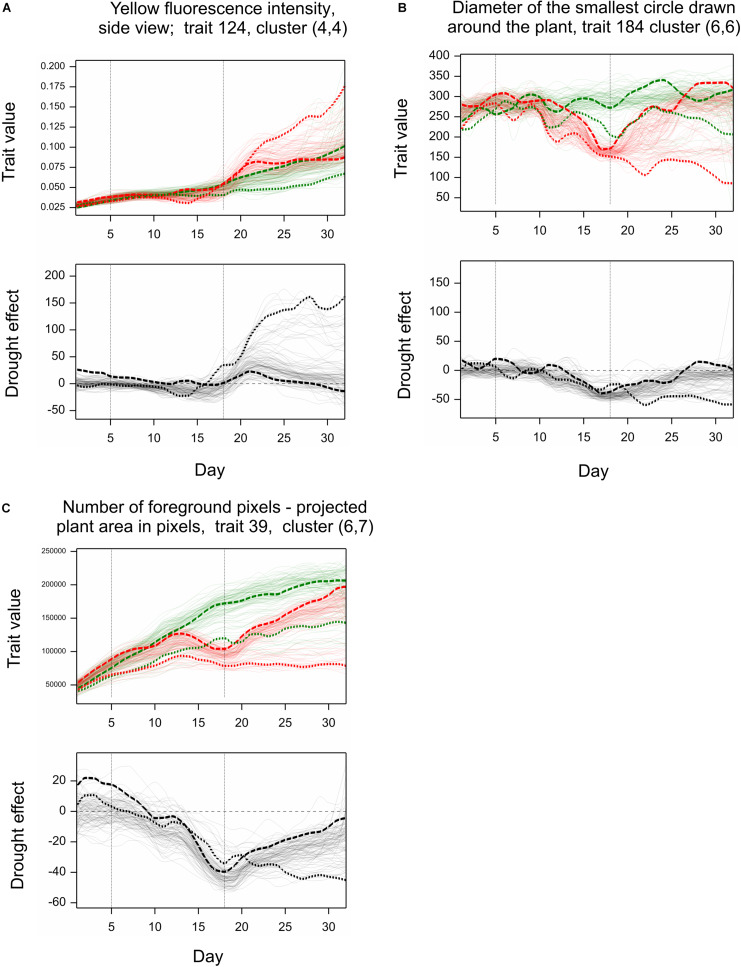
Examples of profiles for image traits observed in time. Top graphs: Observations for all genotypes grown under normal (green) and stress (red) conditions (mean values over two carriers). Bottom graphs: Relative drought effects for all genotypes. Line LA – dashed line, LB – dotted line. **(A–C)** Show profiles for three image-based traits discussed in the text.

Simultaneous classification of traits with respect to time profiles of both drought effects and genetic correlation provided 21 combined clusters (Table 3). Among clusters with a moderate or a large number of traits, one can note cluster (4, 4) of 14 traits characterized by positive drought effects, no recovery and genetic correlation decreasing after drought period, approaching -1 during recovery, and vanishing at the end of observations. All traits in this cluster were obtained from fluorescent images and describe color (eight traits, yellow or red fluorescence intensity – positive effect means more red or yellow; color or brightness uniformity – positive effect means less uniform) or texture of plants (six traits, positive effect means coarser surface). Profiles of mean values and drought effects for an exemplary trait from this group, trait no. 124 (yellow fluorescence intensity), for all lines, are shown in Figure 4A; characteristic is a late appearance of positive drought effects (increased yellowness in drought), no recovery for most of the lines, and differentiation of the effects among lines leading to decreased genetic correlation between conditions. We also note cluster (6, 6) of 36 traits with negative drought effects, but with no evident decrease of genetic correlation (all traits in this group but one are geometric ones, measured in fluorescent or visible light by top view camera); profiles for a trait from this group, no. 184 (diameter of the smallest circle drawn around the plant), shown in Figure 4B, show that the drought effects were relatively homogeneous for lines, which led to a rather large genetic correlation. The most numerous classes, clusters (5, 7) enriched in texture traits and (6,7) enriched in geometric traits, contain traits with moderate drought effects and no interaction of genotypes and environment.

**TABLE 3:** Numbers of traits in simultaneous classification with respect to the time profile of mean drought effect and genetic correlation between conditions.

**Clustering criterion**		**Genetic correlation cluster**							
		**1**	**2**	**3**	**4**	**5**	**6**	**7**	**Total**
	1	2	0	2	0	0	0	0	4
	2	0	0	0	0	4	0	0	4
	3	0	0	0	2	2	0	2	6
Mean drought	4	0	0	0	14	5	4	3	26
effect cluster	5	1	5	5	12	21	10	62	116
	6	0	0	5	0	2	36	87	130
	Total	3	5	12	28	34	50	154	286

*Cluster numbers correspond to the ones shown in Figure 3.*

The behavior of lines LA and LB with respect to traits shown in Figures 4A–C can be described as follows. The (relatively) less resistant line LA showed a larger “yellowing” effect [trait 124, cluster (4,4)] during drought (days 13–14) than the more resistant line LB; after drought, the effects for LA were much smaller, and LA, unlike LB, showed recovery by the last day of observations. The smallest circle diameter [trait 184, cluster (6,6)] was higher for LA than for LB both in control and drought conditions, and the negative drought effects (smaller diameter in drought), starting during the drought period, were initially larger for LB, then similar for both lines, and, after drought, indicated a reduced recovery for LB. The projected plant area [trait 39 in Figure 4C, cluster (6,7)] was larger for LA than for LB; the negative drought effects for LA was bigger than for LB on days 14–20, but then was smaller, with, again, no recovery for LB. So, for two of the presented traits (124, 39), the reaction of LA during drought could be stronger than that of LB, but the difference between LA and LB was more visible during the re-watering period than during drought.

**FIGURE 5:**
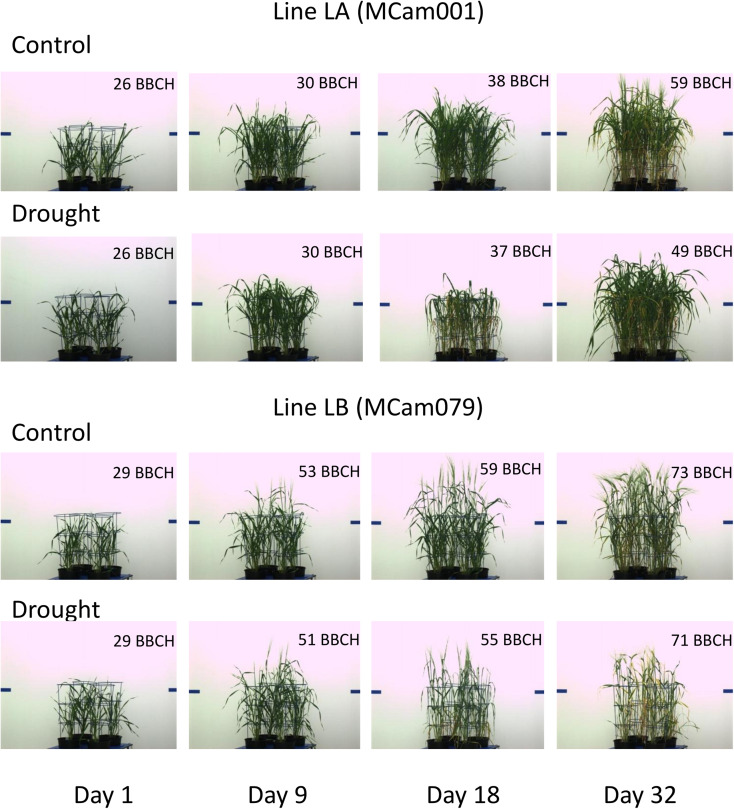
Side view VIS images of lines LA and LB on selected days of experiment.

The above characteristics are further illustrated by selected side view VIS images of lines LA and LB shown in Figure 5. For example, a good recovery for LA and its lack for LB with respect to projected plant area is well-visible. The pictures show differences in developmental stages between lines (with approximate BBCH ratings; see section “Discussion”). LB has reached heading during the drought period; this process affected its behavior with respect to many traits, and could cause apparently smaller drought effects in terms of yellowing (trait 124) or plant area loss (trait 39). LA has started heading at the end of drought period, when heading in LB was completed; this explains smaller drought effects for LA immediately after drought. During re-watering, LA continued heading and showed recovery, whereas LB, being at the flowering stage and going toward maturity, did not show signs of recovery.

### Correlations Between Traits in Time and Post-harvest Traits

After computing – for each day of experiment – correlation coefficients between relative drought effects for traits observed in time and for traits observed after harvesting, the numbers of days were counted on which the correlation was significant, and these counts were summed up over time traits (Table 4).

**TABLE 4:** Numbers of days at which drought effects for traits measured after harvesting were significantly correlated with drought effects for a trait measured in time (significance of correlations declared at *P* < 0.001).

**Trait no.**	**Symbol**	**Trait name**	**Number of days with positive correlations**	**Number of days with negative correlations**	**Total number of days with correlations**
1	PH	Plant height (cm)	10	3	13
2	NTP	Number of productive tillers	4	7	11
3	NTT	Total number of tillers	19	19	38
4	TGW	1000-grain weight (g)	230	111	341
5	LSm	Length of main spike (cm)	17	113	130
6	NSm	Number of spikelets per main spike	193	505	698
7	NGm	Number of grains per main spike	103	297	400
8	GWm	Grain weight per main spike (g)	237	481	718
9	LSl	Length of lateral spike (cm)	35	26	61
10	NSl	Number of spikelets per lateral spike	23	37	60
11	NGl	Number of grains per lateral spike	28	20	48
12	GWl	Grain weight per lateral spike (g)	411	323	734

The total numbers of “days with correlation” were the highest for traits describing properties of main spike, GWl and TGW. The lowest numbers were obtained for plant height and tiller counts. Taking into account this information, and the information on correlations of RDE for traits observed after harvesting (section “Analysis of Post-harvest Traits”), the following detailed analyses were chosen for interpretation of relationships between time-course and after-harvest phenotyping:

a) Analysis of correlations between traits measured in time and the (correlated complex of) traits describing main spikes – traits no. 5–8; the correlation profiles were averaged over four after-harvest traits taken into account,
b) Analysis of correlations between traits measured in time and TGW.

**FIGURE 6:**
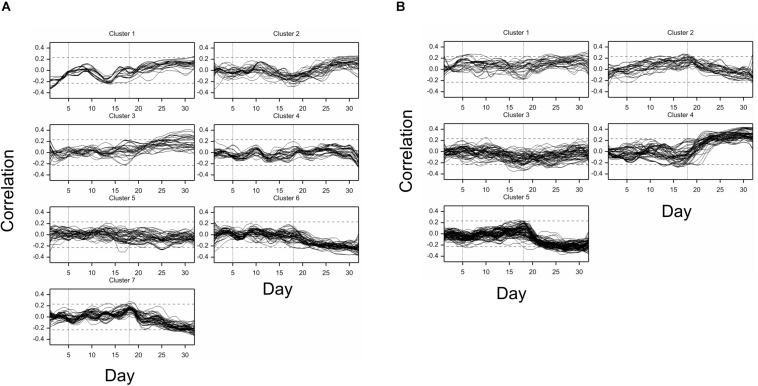
Time profiles of correlations of drought effects (DE) for traits measured in time with **(A)** DE for main spike traits measured after harvest (correlations averaged over traits 5–8), and **(B)** DE for thousand grain weight, clustered using minimum within-group sum of squares (WSS) criterion. Smooth lines drawn using splines with 16 d.f., vertical dashed lines mark start and end of the drought treatment period.

For case (a), clustering of profiles of correlation coefficients (Figure 6A) revealed seven relatively homogeneous clusters, representing various time profiles of correlation; in some clusters, a trend in time is evident despite the fact that some correlations are not statistically significant (but note that smoothed profiles were drawn, with correlations on particular days possibly significant). Correlations decreasing or increasing during or immediately after drought, and then taking opposite values, were observed in, correspondingly, clusters 1 (27 fluorescent traits) and 2 (32 traits; 53% in color traits, 25% RGB), and 7 (58 traits; 81% geometric traits); or approximately constant until a few days after the end of drought period, and then going up or down, in, correspondingly, clusters 3 (32 traits; no overrepresentation) and 6 (57 traits; 75% top view). Clusters 4 (36 traits; 97% geometric, 100% side view) and 5 (44 traits) were made of traits with rather low and constant correlation with RDE for traits describing main spikes.

For case (b), grouping of correlation profiles provided five clusters of traits (Figure 6B), with clusters 4 (63 traits; 65% fluorescent; 41% texture) and 5 (102 traits; 77% geometric) containing features characterized by, correspondingly, decreasing and increasing correlation in drought period, and going to opposite values afterward. Cluster 2 contained 33 traits (90% geometric), for which correlations with TGW increased during the drought period and decreased afterward.

By merging clusters characterized by similar trends in time: (1,2,3), (6,7), (4,5) for correlations with main spike traits, and (4), (2,5), (1,3) for correlations with TGW, we obtained the less fine divisions of traits (Table 5). For most of the traits, the profiles of correlations with spike traits and with TGW had similar characteristics (both increasing, both decreasing or both constant – the diagonal in Table 5). The joint cluster of traits which did not correlate with main spike traits but revealed a correlation with TGW decreasing to negative values contained 25 traits (92% geometric, 80% side view). In addition to correlations of drought effects, we also computed correlations between absolute values of traits measured in time and absolute values of traits measured after harvesting (Supplementary Table 3). Comparing these results with those shown in Table 4, we can see that total numbers of “days with correlation” were larger for absolute trait values than for RDE for plant height under drought (546 v. 13), for total number of tillers under control conditions (404 v. 19), and – considerably – for all traits characterizing main and lateral spikes, both under optimal and limited irrigation (1305–5935 v. 48–734). A reverse situation was observed for TGW, where the days with correlation were less numerous for absolute trait values (230 in control conditions, 235 under drought) than for relative drought effects (341).

**TABLE 5:** Classification of traits with respect to profiles of correlation of their RDE with RDE of after-harvest traits: number of traits and overrepresented categories of traits (in %).

		**Clusters for correlation with TGW**		
		**4 increasing**	**2, 5 decreasing**	**1, 3 constant**
Clusters for correlation with main spike traits	1, 2, 3 increasing	62, 64% fluor, 53% color, 40% texture	8	21, 38% hsv
	6, 7 decreasing	0	102, 81% geometric, 64% top	13
	4, 5 constant	1	25, 92% geometric, 80% side	54, 83% side

### Selection of Traits Based on Correlations of RDE

The joint classification of 286 traits observed in time with respect to the shape of profiles of mean drought effects and of genetic correlations between conditions (section “Image-Based Phenotyping of the Dynamics in Drought Response”), and shape of the profiles of correlations with after-harvest traits (section “Correlations Between Traits in Time and Post-harvest Traits”), provided: 59 groups of traits in case of correlation with spike traits, and 46 groups in case of correlation with TGW.

**TABLE 6:** Image-based traits characterized by significant and frequent (over the time-course of phenotyping) correlations with after-harvest traits describing spike traits (21 selected) and thousand grain weight (11 selected).

**Trait no.**	**Trait name**	**Trait description**	**Selected for spike traits**	**Selected for TGW**
16	hull.circularity.geometry.trait.based. on.fluorescence.side.view.	Indicates similarity of the convex hull to a circle, ranges between 0 and 1. A circular object has value 1.	Yes	Yes
39	area.geometry.trait.based.on. visible.light.side.view.px.2.	Number of foreground pixels. Therefore, projected plant area in pixels.		Yes
75	hull.pc2.geometry.trait.based.on. visible.light.side.view.px.	If a line connects the two most far from each other situated plant pixels is drawn, this number indicates the sum of the maximum distances of other plant pixels from the left and right of this line.	Yes	Yes
104	hsv.h.mean.color.related.trait.based. on.fluorescence.side.view.	Mean – first order texture property (independent of pixel neighbors). Calculated on grayscale image derived from channel Hue (HSV).	Yes	Yes
115	hsv.v.mean.color.related.trait.based. on.fluorescence.side.view.	Mean – first order texture property (independent of pixel neighbors). Calculated on grayscale image derived from channel Brightness (HSV).		Yes
124	intensity.phenol.mean.color.related. trait.based.on.fluorescence.side.view.	A relative indicator of the yellow fluorescence intensity, not taking into account brightness but only the color hue (red = no intensity, yellow = high intensity). Detailed information will be added to the documentation.	Yes	
135	lab.b.stddev.color.related.trait.based.on. fluorescence.side.view.	The standard deviation of the b values in the L*a*b* color space of the plant pixels. The lower this value, the more uniform is the plant color		Yes
139	hsv.h.yellow2green.color.related.trait. based.on.visible.light.side.view.	Proportion of yellow color plant pixels (histogram bin 3) divided by the count of green color pixels (bins 4–7). This value is only valid if the bin count has not been changed from 20, otherwise the involved bins represent different colors.	Yes	
141	hsv.h.skewness.color.related.trait. based.on.visible.light.side.view.	The “skewness” of the hue values of the plant pixels. “skewness” is a statistical term, indicating the tendency of the value distribution to lean to one side of the value range. The documentation will include a more complete description of this trait in the future; see reference literature for full details.	Yes	
143	hsv.h.mean.color.related.trait.based. on.visible.light.side.view.	Mean – first order texture property (independent of pixel neighbors). Calculated on grayscale image derived from channel Hue (HSV).	Yes	
144	hsv.h.brown2green.color.related.trait. based.on.visible.light.side.view.	Proportion of brown color plant pixels (histogram bin 2) divided by the count of green color pixels (bins 4–7). This value is only valid if the bin count has not been changed from 20, otherwise the involved bins represent different colors.	Yes	Yes
150	lab.a.stddev.color.related.trait.based. on.visible.light.side.view.	The standard deviation of the a-values in the L*a*b* color space of the plant pixels. The lower this value, the more uniform is the plant color.	Yes	
154	hsv.v.skewness.color.related.trait. based.on.visible.light.side.view.	The “skewness” of the brightness values of the plant pixels. “Skewness” is a statistical term, indicating the tendency of the value distribution to lean to one side of the value range. The documentation will include a more complete description of this trait in the future; see reference literature for full details.	Yes	
157	rgb.red.mean.color.related.trait. based.on.visible.light.side.view.	Average intensity of the red channel of the plant pixels in the visible light image.	Yes	
166	rgb.g.std.texture.trait.based.on. fluorescence.side.view.	Standard Deviation – first order texture property (independent of pixel neighbors). Calculated on grayscale image derived from channel Green (RGB).		Yes
174	rgb.r.std.texture.trait.based.on. fluorescence.side.view.	Standard Deviation – first order texture property (independent of pixel neighbors). Calculated on grayscale image derived from channel Red (RGB).	Yes	
194	hull.area.zoom.corrected.geometry.trait. based.on.fluorescence.top.view.mm.2.	Normalized area (in real-world coordinates) of the convex hull, which is the shortest convex line drawing around the plant.	Yes	
256	hull.compactness.16.geometry.trait. based.on.visible.light.top.view.	borderPixels * borderPixels/filledArea (all of convex hull)	Yes	Yes
261	leaf.length.sum.geometry.trait. based.on.visible.light.top.view.px.	Skeleton length.	Yes	
269	hull.pc2.geometry.trait.based.on. visible.light.top.view.px.	If a line connects the two most far from each other situated plant pixels is drawn, this number indicates the sum of the maximum distances of other plant pixels from the left and right of this line.	Yes	
316	ndvi.color.related.trait.based.on. multi.camera.top.view.	ndvi = (averageNir – averageVisR)/(averageNir + averageVisR)	Yes	
320	hsv.v.mean.color.related.trait. based.on.visible.light.top.view.	Mean – first order texture property (independent of pixel neighbors). Calculated on grayscale image derived from channel Brightness (HSV).	Yes	
321	hsv.s.stddev.color.related.trait. based.on.visible.light.top.view.	The standard deviation of the saturation values of the plant pixels. The lower this value, the more uniform is the saturation of the plant colors.		Yes
325	hsv.h.stddev.color.related.trait. based.on.visible.light.top.view.	The standard deviation of the hue values of the plant pixels. The lower this value, the more uniform is the plant color.		Yes
330	lab.b.stddev.color.related.trait. based.on.visible.light.top.view.	The standard deviation of the b values in the L*a*b* color space of the plant pixels. The lower this value, the more uniform is the plant color	Yes	
337	rgb.red.mean.color.related.trait. based.on.visible.light.top.view.	Average intensity of the red channel of the plant pixels in the visible light image.	Yes	
353	rgb.b.std.texture.trait.based.on. visible.light.top.view.	Standard Deviation – first order texture property (independent of pixel neighbors). Calculated on grayscale image derived from channel Blue (RGB).	Yes	

*Correlations computed for drought effects.*

The final selection of traits observed in time during drought stress, with various characterizations with respect to time-course of mean relative drought effects, genetic correlation (GE interaction), and correlation with after-harvest traits, was made by finding in all joint categories defined above the time traits characterized by the number of correlation days maximal in the given category and bigger than the upper quartile of the distribution of number of correlation days for all traits. This operation provided the list of 21 image-derived traits for which RDE were highly correlated with RDE for spike traits, and 11 traits with the same property but with respect to TGW (Table 6 and Supplementary Table 4). The set of time traits selected on the basis of correlation with both main spike traits and TGW consists of traits characterizing: plant convex hull circularity (16), plant’s convex hull geometry (75), texture (104), proportion of brown pixels in relation to green pixels (144), and hull compactness (256). Characteristics of these traits are summarized in Supplementary Table 5.

### Prediction of RDE for Post-harvest Traits Based on Image Phenotyping During Drought

**FIGURE 7:**
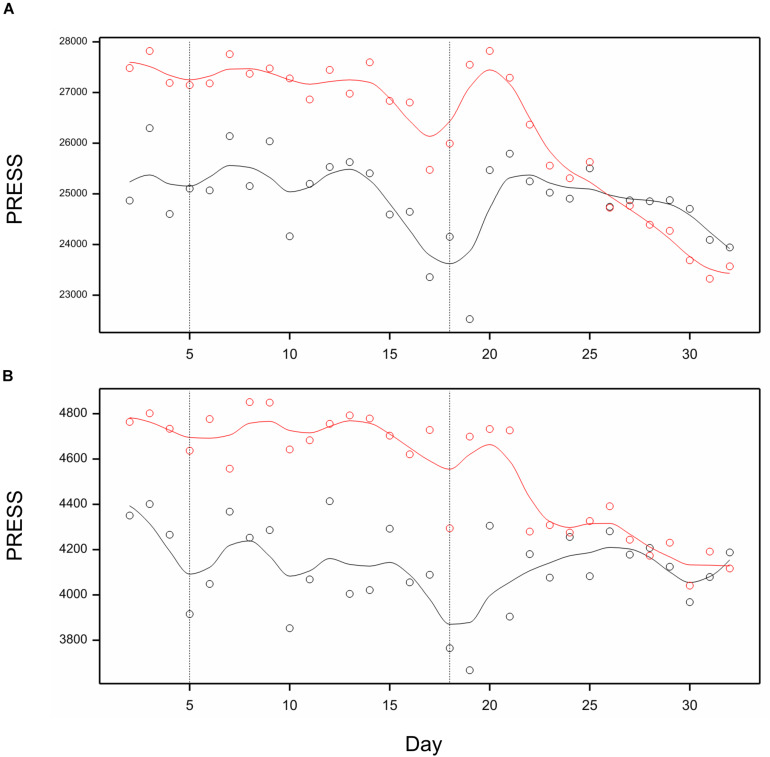
Profiles of Predictive Residual Error Sum of Squares (PRESS) for PLS regression of RDE for postharvest traits on RDE for time traits, on days 2, 3, …, 32. **(A)** PLS for main spike traits as dependent variables. **(B)** PLS for trait TGW as a dependent variable. Black lines: prediction by all image-based traits; red lines – prediction by 22 traits selected for spike in **(A)** and 12 traits selected for TGW in **(B)**. Lines smoothed by splines (12 df).

Utilizing image-based traits selected in previous section, we performed partial least-squares regression model fitting to predict RDE for after-harvest traits, separately for each day of observations. The profiles of the measure of model fit, predictive residual error sum of squares (PRESS), for prediction of RDE for four main spike traits using RDE of 21 image traits are shown in Figure 7A. The prediction quality was relatively good when traits measured on days 15–20 were used. Toward the end of imaging period, prediction by 21 selected traits was better than by using all traits. For prediction of TGW, the profile of PRESS corresponding to 11 selected traits shows a steady improvement, whereas the profile for all traits – a temporary one on days 18–19 (Figure 7B). On days 31–32, quality of prediction for both spike traits and TGW using the selected subset of traits is as good or better than prediction using all 286 traits.

**FIGURE 8:**
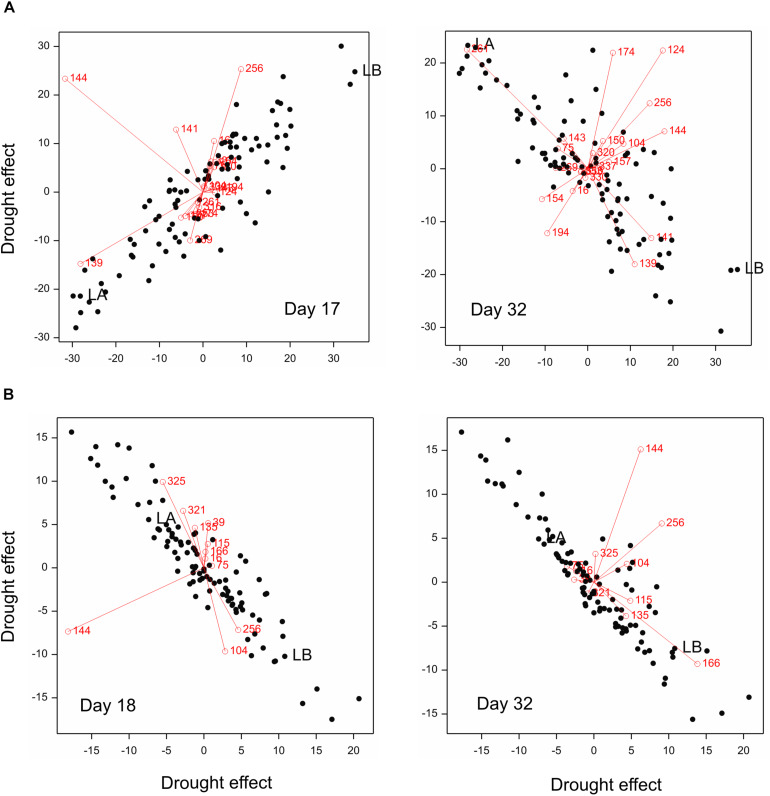
PLS biplots for **(A)** main spike traits and **(B)** TGW, with explanatory variables on days 17, 18, and 32 (variables in PLS regression: *Y* = main spike traits or TGW; *X* = selected traits – numbers of traits shown in the plot). RILs marked: LA (MCam001) – relatively susceptible to drought, LB (MCam079) – relatively resistant.

The image-based traits with a dominant role in prediction can be identified in PLS biplots shown in Figure 8. Genotypes more resistant to drought in terms of main spike characteristics (namely, losing less main spike length and grains) were characterized:

– On day 17 (when a local minimum of PRESS was observed) – by smaller positive drought effects for image trait 139 (i.e., relatively smaller – in comparison to other genotypes – increase of number of yellow pixels in relation to green ones) and by bigger positive effects for traits 256 (bigger loss of compactness),
– On day 32 (at the end of imaging) – by bigger positive drought effects for image traits 139 (bigger increase of number of yellow pixels in relation to green) and 141 (bigger shift of distribution of hue values to the left, i.e., toward red values), and bigger negative effects of trait 261 (bigger loss of skeleton length, i.e., greater reduction of the shoot length).

Genotypes relatively resistant in terms of TGW (losing less) were characterized:

– On day 18 (local minimum) – by bigger positive effects of traits 256 (bigger loss of hull compactness) and smaller positive effects of trait 325 (smaller increase of color non-uniformity),
– On day 32 (end of imaging) – by bigger positive effects for trait 166 (bigger increase of coarseness of the plant surface).

The above relationship can be also confirmed by comparing the behavior of lines LA (relatively susceptible) and LB (relatively resistant) with respect to above-mentioned image traits (Supplementary Figure 3).

## Discussion

This paper discusses the employment of high-throughput, non-invasive imaging platform to the characterization of phenotypic reaction of barley lines to limited irrigation. We analyzed the data set provided by the image analysis pipeline containing observations of more than 350 traits with the aim of defining a non-redundant set of image-derived phenotypic characteristics useful for a further inference on the fitness of genotypes. The criteria for selection of traits were based on genetic considerations and on relating the picture of RILs observed during drought stress to their characteristics with respect to yield-related traits observed after harvesting.

### Analysis of Post-harvest Traits

In our experiment, variance components for yield-related traits of RILs were lower under drought than well-watered conditions. This result confirms observations from practical breeding that differences between genotypes (lines/cultivars) in crop performance features are more evident in favorable conditions than under stress. That is widely used in breeding high-yielding cultivars which are selected most often under optimal conditions (Laingi and Fischer, 1977; Atlin et al., 2017). Genetic correlations between conditions estimated in our experiment showed that the strategy of selection for any environment may work better for traits characterizing spike architecture than for yield or TGW, and will not work for plant architecture (number of tillers).

Our inference was based not on absolute trait values, but on relative drought effects characterizing the reaction of genotypes to water shortage. We utilized these effects for four characteristics of main spikes to identify genotypes with a relative good (like LB) and poor (like LA) resistance to water shortage. We attribute the presence of such genotypes in the studied population to the properties of its parents: Maresi, a good-quality European variety, and Cam/B1/CI, a line of Asian origin. The characterization by relative effects is independent of the fact that LB (and, indeed, Cam/B1/CI) behaved much worse than LA (and Maresi) in optimal conditions.

### Phenotyping in Time

The analysis of image-derived data acquired over 32 days of the experiment run on the high-throughput phenotyping platform provided information on the behavior of genotypes during drought period and during re-watering. Low replicability of observations obtained from infrared imaging was revealed; this is in contrast with findings of Chen et al. (2014), who reported good reproducibility of NIR-based traits. Relative drought effects (mean – averaged over all genotypes; positive or negative – depending on the trait nature) were observed for almost all features extracted by the image analysis pipeline; they started to be significant on the fourth day of limited watering. The earliest and largest negative effects were observed for plant area measured in visible or fluorescent light by side-view camera. The largest positive effects, starting in the middle of drought period (similarly as in Chen et al., 2014), were noted for proportions of yellow, red or brown pixels measured in visible light by side- and top-view cameras. So, the first observable effect of reduced watering was the slowed down, and then strongly reduced, plant body development; it was followed by plants turning less green as a result of chlorophyll degradation (Ghandchi et al., 2016). Reducing the chlorophyll content causes a decrease in photosynthesis intensity and thus limits the growth and development of plants (Buschmann and Lichtenthaler, 1998; Buschmann et al., 2000; Jansen et al., 2009).

In addition to relative drought effects, we studied the time profiles of genetic variances and of genetic correlation coefficients between conditions, which informed us about the variability and co-variability (GE interaction) of the traits under different conditions. The genetic variance under drought was bigger than under control conditions in the re-watering period (especially for many texture traits). This is in contrast with what was observed for after-harvest phenotypic traits and suggests that drought resistant genotypes might be selected under limited watering using properly chosen image-derived traits. This possibility is further confirmed by the existence of many traits (especially those measuring color or texture) for which the GE interaction increased toward the end of the observation period.

The simultaneous classification of traits with respect to time profiles of drought effects and of genetic correlation between drought and control showed that numerous traits describing color or texture of plants were characterized by positive drought effects and decreased correlation. It indicates that under drought the leaves discolored (turned brown or yellow), while under optimal watering they were still green, but the effect sizes were different for different genotypes and the correlation between drought and control decreased. On the other hand, many traits describing geometrical properties of plants, measured in fluorescent or visible light by top view camera, showed negative drought effects, but with no evident decrease of genetic correlation between treatments. This again suggests the use of color and texture traits in preference to geometric ones in selection.

### Relationships Between Traits in Time and Post-harvest Traits

The numbers of days on which the significant correlation coefficients between relative drought effects for traits observed in time and traits observed after harvesting were the highest for spike characteristics and TGW, while the lowest for final number of tillers and plant height. That was due to the duration of drought in the context of plant development. Phenotyping started when plants were at the stage of tillering (26–29 in the BBCH scale). During the period of water stress and 2 weeks after drought, they have undergone the phases of tillering, and shoot and spike development, which may explain observed associations. On the other hand, re-watering contributed to grain filling and the emergence of new shoots which did not form grains. Plants continued growing also after the period of image phenotyping; therefore, there is no clear relationship between image-derived traits and the final number of tillers and plant height. This result suggests that a different drought application scenario would be necessary to study the reaction of genotypes to drought with respect to plant architecture.

About 60 image traits were selected (mainly color and texture) with RDE characterized by correlation with RDE for post-harvest spike traits and TGW increasing in time (to positive values). As we noted, this was due to the fact that the lines classified by us as “relatively resistant,” e.g., LB, did not recover from the drought by the end of the observation period (positive color/texture effect), whereas the “relatively susceptible” lines, e.g., LA, did recover (negative color/texture effect). For example, we have shown that during drought, the leaves of LB turned yellow to a lesser extent than those of LA, but after re-watering, toward the end of observations, the “yellowing” effect of the LB deepened and remained, while for LA, gradually decreased and become even negative at day 32. It indicates that LA has the ability to produce new shoots and green leaves after re-watering, which reduced the overall “yellowing” effect.

On the other hand, about 100 traits (mostly geometric) had RDE characterized by correlation with RDE for post-harvest spike traits and TGW decreasing over time (to negative values). This was also caused by the lack of regeneration of “relatively resistant” genotypes in terms of plant architecture by the end of the observation period. For example, drought caused a reduction in the projected area of plants (trait 20) and skeleton length (trait 261); the effects were similar for LA and LB, or a bit smaller for LB, until approximately day 20, when recovery started for line LA but not for LB.

We can safely assume that the differences explained above between lines LA and LB, more pronounced during re-watering than during drought, originated from the properties of the parents: Cam/B1/CI is a very early line as compared to Maresi, with about 10 days earlier heading and earlier maturity (Mikołajczak et al., 2016). A corresponding difference between lines LB and LA was visible in image phenotyping (see section “The Trait Selection Procedure” for more discussion of phenology). The lack of recovery of LB could be caused by its inability to regenerate after heading, in a later developmental stage leading to maturity, whereas less yellow appearance of LA at the later stages could be caused by spikes and new leaves emerging after drought period. The inference that we could make on the basis of relative drought effects and identification of “relatively” resistant and susceptible genotypes shows important differences in the behavior of genotypes. While LA showed a “stay green” and recovery behavior, LB seemed to promote senescence, potentially with rapid mobilization of resources into grain filling during drought (thus avoiding drought effects by a shorter life cycle). The shorter vegetative phase of LB, with fewer tillers, may cause lower yield in the non-stress condition.

In addition to correlations of relative drought effects, we presented results showing that image-based traits expressing good correlations with absolute values of most of the after-harvest traits could be found. This finding creates a possibility of using the approach presented in this paper for characterization of genotypes better suited for cultivation in optimal or suboptimal conditions. Such analysis is in progress and will be reported elsewhere in the context of utilization of molecular polymorphism data for localization of dynamic quantitative trait loci.

### The Trait Selection Procedure

The classification and selection of traits was based on the assumption that useful image-derived features should provide information on three components of RIL’s characterization: environment (drought) effects, genetic correlation between conditions related directly to genotype-by-environment interaction, and the link between behavior during the stress and the final yielding performance. First two parameters are widely used for static characterization of RIL populations observed in traditional experiments with only after-harvesting phenotyping; they were also used for dynamic description of a smaller plant population by Chen et al. (2014) who imaged 18 barley cultivars under well-watered and limited irrigation conditions. Having observations of post-harvest yield-related traits, we were able to extend this idea to correlations between relative drought effects of two sets of traits. By a multiple use of a simple clustering procedure, we divided the set of all traits into disjoint groups with various characteristics, and, finally, selected representatives of these groups valuable for inference. We think that the described procedure can be used extensively in image phenotyping for an unsupervised selection of representative traits obtained from various existing image analysis pipelines. Pipelines used by image phenotyping platforms are continuously modified, and new pipelines are being developed. Their potential to provide new phenotypic characteristics is almost infinite. In such situation, the presented selection procedure can be useful. Linking this procedure to emerging taxonomies of image-based plant traits (Fahlgren et al., 2015; Das Choudhury et al., 2019) will be beneficial for better annotation of data sets.

In our studies, the applied statistical approaches allowed choosing 21 traits out of 286 observed in time for which drought effects were highly correlated with drought effects for spike traits, and 11 traits with the same property but with respect to TGW. The traits common to these two sets characterized plant convex hull geometry related to the growth habit, and texture, proportion of brown pixels and chlorophyll intensity – features related to the earliness. In the studied population, growth habit was mainly determined by the semi-dwarfing gene from Maresi, while earliness by gene(s) from Cam/B1/CI (Mikołajczak et al., 2016, 2017; Ogrodowicz et al., 2017). Segregation of genes determining these two properties largely influenced variation of all the observed traits, both measured in time and after harvest.

In the context of observed phenological differences between studied parental forms and RILs, the problem arises of the influence of these differences on the results of trait selection and on the conclusions. Due to the absence of image-derived traits annotated directly as corresponding to reaching consecutive developmental stages, we attempted to target this problem by visual scanning of (top view RGB) images to score, for each plant, the day of reaching BBCH49 (the stage when flag leaf sheath opens; all data not presented), and using these data as a covariable in the linear mixed model in which drought effects and variance components for RILs were estimated. The differences between significance of mean drought effects in these two models were quite minor (Supplementary Figures 4A,B); the number of significant drought effects in the ANCOVA model was slightly smaller until day 22, which means that for some traits the difference between control and drought conditions was partially explained by differences in phenology. Bigger differences between two models were observed for fold change of genetic variance between drought and control conditions (Supplementary Figures 4C,D). The proposed trait selection procedure performed using results of the ANCOVA model provided 26 traits, out of which 16 were the same as in the procedure based on ANOVA model reported in Results (Table 6), including all five traits selected for both correlation with spike traits and with TGW. Thus, some effect of the fact that RILs were, at a given time point, at different developmental stages, was observed. This points to a need for the development of image recognition algorithms able to score phenology in an automatic way. This additional analysis also further illustrates the flexibility of the proposed trait selection procedure with respect to the statistical model of the experiment. In reference to the discussion on earliness of parental genotypes and RILs, we note that, on average, lines LB and LA reached BBCH49 on imaging days, correspondingly, 6.8 and 25.0 in control conditions, and on days 5.5 and 26.4 under drought, which is consistent with Figure 5. For parental lines Cam/B1/CI and Maresi, the corresponding values were 4.9 and 26.6 in control conditions, and 3.5 and “more than 32” (after the imaging period) under drought, which, in terms of direction of differences, agrees with the results of Mikołajczak et al. (2016).

The final step of our analysis, prediction of RDE for after-harvest traits by RDE of selected image-based traits, was performed to illustrate a possible application of the trait selection procedure. We do not claim that the PLS regression is the best method to perform such prediction. However, we showed that a good fit of a prediction model can be obtained for a small set of selected traits.

## Conclusion

Image phenotyping allows observation of changes in plants during growth. The results of our experiment show these changes in various aspects: changes in color, texture, and geometrical properties of plants under optimal and water stress conditions, and association of the traits evaluated in time with post-harvest characteristics. In most cases, these changes were expected and they are in line with practical observations. Our results permitted to distinguish features related to the convex hull geometry, texture and proportion of brown pixels for which effects of drought were highly correlated with drought effects for spike traits and TGW. Estimated genetic correlations between drought and well-watered conditions showed that strategy of selection for a broad range of environments may be more effective for traits characterizing spike architecture than for grain yield or thousand grain weight, and can be ineffective in the case of number of tillers. RILs relatively susceptible and tolerant to drought were selected and although the yellowing and area loss effects were somewhat smaller for the latter during drought, after re-watering such effects for the tolerant lines deepened and remained, while for susceptible ones gradually decreased which indicates that in the studied population, tolerant lines escaped drought through accelerated plant development. Further results concerning the described experiment and RIL population will combine the presented barley dynamic phenomic data with high-resolution linkage mapping to illustrate the evolution of the genotype-phenotype relationship in time, under water scarcity. Such approach is relevant to identify genes underlying the response of barley plants to drought.

## Data Availability Statement

The data set containing images and observations of image-derived and post-harvest traits in the MIAPPE-compliant ISA-Tab format is available online (Kuczyńska, 2020).

## Author Contributions

AK, MS, and PK conceived and planned the study. AK, KK, KM, and PO performed the experiment. HĆ-K and PK processed the image-derived and post-harvest data and did statistical analysis. MS, TAd, AK, KM, and PO reviewed the literature and provided biological interpretation. AJ, KW-F, and TAl planned and supervised the experiment on the phenotyping platform. SM processed the image-derived data. All co-authors critically reviewed the manuscript and provided constructive feedback on presentation and interpretation of results.

## Conflict of Interest

The authors declare that the research was conducted in the absence of any commercial or financial relationships that could be construed as a potential conflict of interest.

## Abbreviations

BBCH scale: a system for uniform coding of plant’s growth stages (Lancashire et al.1991)
DAS: day after sowing
FLUOR: static fluorescence signals
GC: genetic correlation
GE: genotype by environment interaction
GWl: grain weight per lateral spike
GWm: grain weight per main spike
IAP: Integrated Analysis Platform (Klukas et al.2014)
LA: line with large trait values under control conditions and a large relative loss under drought
LB: line with low trait values under control and a small relative loss under drought
LSl: length of lateral spike
LSm: length of main spike
NGl: number of grains per lateral spike
NGm: number of grains per main spike
NIR: near infrared light spectrum
NSl: number of spikelets per lateral spike
NSm: number of spikelets per main spike
NTP: number of productive tillers
NTT: total number of tillers
PLS: partial least squares
PRESS: predictive residual error sum of squares
RDE: relative drought effects
REML: residual maximum likelihood method
RIL(s): recombinant inbred line(s)
TGW: 1000-grain weight
VIS: visible light spectrum
WSS: within-group sum of squares.

## References

[B1] Atlin G. N. Cairns J. E. Das B. (2017). Rapid breeding and varietal replacement are critical to adaptation of cropping systems in the developing world to climate change. *Science* 12 31–37. 10.1016/j.gfs.2017.01.008 28580238 PMC5439485

[B2] Buschmann C. Langsdorf G. Lichtenthaler H. K. (2000). Imaging of the blue, green, and red fluorescence emission of plants: an overview. *Photosynthetica* 38 483–491. 10.1023/A:1012440903014

[B3] Buschmann C. Lichtenthaler H. K. (1998). Principles and characteristics of multi-colour fluorescence imaging of plants. *J. Plant Physiol.* 152 297–314. 10.1016/S0176-1617(98)80144-2

[B4] Busemeyer L. Mentrup D. Möller K. Wunder E. Alheit K. Hahn V. (2013). Breed vision — a multi-sensor platform for non-destructive field-based phenotyping in plant breeding. *Sensors* 13 2830–2847. 10.3390/s130302830 23447014 PMC3658717

[B5] Cabrera-Bosquet L. Crossa J. von Zitzewitz J. Serret M. D. Luis Araus J. (2012). High-throughput phenotyping and genomic selection: the frontiers of crop breeding convergeF. *J. Integr. Plant Biol.* 54 312–320. 10.1111/j.1744-7909.2012.01116.x 22420640

[B6] Cattivelli L. Rizza F. Badeck F.-W. Mazzucotelli E. Mastrangelo A. M. Francia E. (2008). Drought tolerance improvement in crop plants: an integrated view from breeding to genomics. *F. Crop. Res.* 105 1–14. 10.1016/J.FCR.2007.07.004

[B7] Ceccarelli S. (1994). Specific adaptation and breeding for marginal conditions. *Euphytica* 77 205–219. 10.1007/BF02262633

[B8] Ceccarelli S. Grando S. (1999). “Barley landraces from the fertile crescent: a lesson for plant breeders,” in *Genes in the Field: On-Farm Conservation Of Crop Diversity*, ed. Brush S. B. (Boca Raton, FL: IPGRI), 51–76.

[B9] Chen D. Neumann K. Friedel S. Kilian B. Chen M. Altmann T. (2014). Dissecting the phenotypic components of crop plant growth and drought responses based on high-throughput image analysis. *Plant Cell* 26 4636–4655. 10.1105/tpc.114.129601 25501589 PMC4311194

[B10] Das Choudhury S. Samal A. Awada T. (2019). Leveraging image analysis for high-throughput plant phenotyping. *Front. Plant Sci.* 10:508. 10.3389/fpls.2019.00508 31068958 PMC6491831

[B11] Fahlgren N. Gehan M. A. Baxter I. (2015). Lights, camera, action: high-throughput plant phenotyping is ready for a close-up. *Curr. Opin. Plant Biol.* 24 93–99. 10.1016/J.PBI.2015.02.006 25733069

[B12] Famoso A. N. Clark R. T. Shaff J. E. Craft E. McCouch S. R. Kochian L. V. (2010). Development of a novel aluminum tolerance phenotyping platform used for comparisons of cereal aluminum tolerance and investigations into rice aluminum tolerance mechanisms. *Plant Physiol.* 153 1678–1691. 10.1104/pp.110.156794 20538888 PMC2923895

[B13] Ghandchi F. P. Caetano-Anolles G. Clough S. J. Ort D. R. (2016). Investigating the control of chlorophyll degradation by genomic correlation mining. *PLoS One* 11:e0162327. 10.1371/journal.pone.0162327 27618630 PMC5019398

[B14] Goulden C. H. (1939). “Problems in plant selection,” in *Proceedings of the Seventh Genetices Congfiguration*, Edinburgh.

[B15] Grando S. von Bothmer R. Ceccarelli S. (2001). “Genetic diversity of barley: use of locally adapted germplasm to enhance yield and yield stability of barley in dry areas,” in *Broadening the Genetic Base Of Crop Production*, ed. Cooper H. D. (New York, NY: CABI), 351–372.

[B16] Grubbs F. E. (1950). Sample criteria for testing outlying observations. *Ann. Math. Stat.* 21 27–58. 10.1214/aoms/1177729885

[B17] Hartmann A. Czauderna T. Hoffmann R. Stein N. Schreiber F. (2011). HTPheno: an image analysis pipeline for high-throughput plant phenotyping. *BMC Bioinformatics* 12:148. 10.1186/1471-2105-12-148 21569390 PMC3113939

[B18] Honsdorf N. March T. J. Berger B. Tester M. Pillen K. (2014). High-throughput phenotyping to detect drought tolerance QTL in wild barley introgression lines. *PLoS One* 9:e97047. 10.1371/journal.pone.0097047 24823485 PMC4019662

[B19] Jansen M. Gilmer F. Biskup B. Nagel K. A. Rascher U. Fischbach A. (2009). Simultaneous phenotyping of leaf growth and chlorophyll fluorescence via GROWSCREEN FLUORO allows detection of stress tolerance in *Arabidopsis thaliana* and other rosette plants. *Funct. Plant Biol.* 36 902 10.1071/FP09095 32688701

[B20] Jones P. D. Lister D. H. Jaggard K. W. Pidgeon J. D. (2003). Future climate impact on the productivity of sugar beet (*Beta vulgaris* L.) in Europe. *Clim. Change* 58 93–108. 10.1023/A:1023420102432

[B21] Junker A. Muraya M. M. Weigelt-Fischer K. Arana-Ceballos F. Klukas C. Melchinger A. E. (2015). Optimizing experimental procedures for quantitative evaluation of crop plant performance in high throughput phenotyping systems. *Front. Plant Sci.* 5:770. 10.3389/fpls.2014.00770 25653655 PMC4299434

[B22] Klukas C. Chen D. Pape J. M. (2014). Integrated analysis platform: an open-source information system for high-throughput plant phenotyping. *Plant Physiol.* 165 506–518. 10.1104/pp.113.233932 24760818 PMC4044849

[B23] Kuczyńska A. (2020). Phenotypic image data of spring barley (Hordeum vulgare L.) RIL population under drought. e!DAL - Plant Genomics and Phenomics Research Data Repository (PGP), IPK Gatersleben, Seeland OT Gatersleben, Corrensstraße 3, 06466, Germany. 10.5447/IPK/2020/14

[B24] Laingi D. R. Fischer R. A. (1977). Adaptation of semidwarf wheat cultivars to rainfed conditions. *Euphytica* 26 129–139. 10.1007/BF00032078

[B25] Lancashire P. D. Bleiholder H. van den Boom T. Langeluddeke P. Strauss R. Weber E. (1991). A uniform decimal code for growth stages of crops and weeds. *Ann. Appl. Biol.* 119 561–601. 10.1111/j.1744-7348.1991.tb04895.x

[B26] Mikołajczak K. Kuczyńska A. Krajewski P. Sawikowska A. Surma M. Ogrodowicz P. (2017). Quantitative trait loci for plant height in Maresi×CamB barley population and their associations with yield-related traits under different water regimes. *J. Appl. Genet.* 58 23–35. 10.1007/s13353-016-0358-1 27447461 PMC5243891

[B27] Mikołajczak K. Ogrodowicz P. Gudyś K. Krystkowiak K. Sawikowska A. Frohmberg W. (2016). Quantitative trait loci for yield and yield-related traits in spring barley populations derived from crosses between european and syrian cultivars. *PLoS One* 11:e0155938. 10.1371/journal.pone.0155938 27227880 PMC4881963

[B28] Munns R. James R. A. Sirault X. R. R. Furbank R. T. Jones H. G. (2010). New phenotyping methods for screening wheat and barley for beneficial responses to water deficit. *J. Exp. Bot.* 61 3499–3507. 10.1093/jxb/erq199 20605897

[B29] Muraya M. M. Chu J. Zhao Y. Junker A. Klukas C. Reif J. C. (2017). Genetic variation of growth dynamics in maize (*Zea mays* L) revealed through automated non-invasive phenotyping. *Plant J.* 89 366–380. 10.1111/tpj.13390 27714888

[B30] Neumann K. Zhao Y. Chu J. Keilwagen J. Reif J. C. Kilian B. (2017). Genetic architecture and temporal patterns of biomass accumulation in spring barley revealed by image analysis. *BMC Plant Biol.* 17:137. 10.1186/s12870-017-1085-4 28797222 PMC5554006

[B31] Ogrodowicz P. Adamski T. Mikołajczak K. Kuczyńska A. Surma M. Krajewski P. (2017). QTLs for earliness and yield-forming traits in the Lubuski × CamB barley RIL population under various water regimes. *J. Appl. Genet.* 58 49–65. 10.1007/s13353-016-0363-4 27503092 PMC5243898

[B32] Paulus S. Dupuis J. Riedel S. Kuhlmann H. (2014). Automated analysis of barley organs using 3D laser scanning: an approach for high throughput phenotyping. *Sensors* 14 12670–12686. 10.3390/s140712670 25029283 PMC4168454

[B33] R Core Team (2018). *R: A Language And Environment For Statistical Computing.* Vienna: R Foundation for Statistical Computing.

[B34] Sanchez A. C. Subudhi P. K. Rosenow D. T. Nguyen H. T. (2002). Mapping QTLs associated with drought resistance in sorghum (*Sorghum bicolor* L. *Moench*). *Plant Mol. Biol.* 48 713–726. 10.1023/A:1014894130270 11999845

[B35] Tuberosa R. (2012). Phenotyping for drought tolerance of crops in the genomics era. *Front. Physiol.* 3:347. 10.3389/fphys.2012.00347 23049510 PMC3446691

[B36] VSN International (2013). *GenStat for Windows*, 16th Edn, Hemel Hempstead: VSN International.

