# Supplementary material for: Image Phenotyping of Spring Barley (Hordeum vulgare L.) RIL Population Under Drought: Selection of Traits and Biological Interpretation
Source: Front Plant Sci. 2020 Jun 9;11:743. doi: 10.3389/fpls.2020.00743 (PMC7296146; doi:10.3389/fpls.2020.00743)
Supplement: Supplementary file 3 [file Data_Sheet_1.docx]

Supplementary Table 3. Numbers of days at which absolute values of traits measured after harvesting were significantly correlated with traits measured in time (significance of correlations declared at P < 0.001).

| Trait no. | Trait name | Traits observed in control conditions | | | Traits observed under limited irrigation | | |
| --- | --- | --- | --- | --- | --- | --- | --- |
|  |  | Number of days with positive correlations | Number of days with negative correlations | Total number of days with correlations | Number of days with positive correlations | Number of days with negative correlations | Total number of days with correlations |
| 1 | Plant height (cm) | 7 | 1 | 8 | 278 | 268 | 546 |
| 2 | Number of productive tillers | 6 | 0 | 6 | 0 | 0 | 0 |
| 3 | Total number of tillers | 129 | 275 | 404 | 23 | 23 | 46 |
| 4 | 1000-grain weight (g) | 203 | 27 | 230 | 78 | 157 | 235 |
| 5 | Length of main spike (cm) | 1125 | 448 | 1573 | 939 | 1019 | 1958 |
| 6 | Number of spikelets per main spike | 3449 | 2213 | 5662 | 2514 | 2466 | 4980 |
| 7 | Number of grains per main spike | 3462 | 2235 | 5697 | 1772 | 1720 | 3492 |
| 8 | Grain weight per main spike (g) | 3142 | 1967 | 5109 | 1416 | 1679 | 3095 |
| 9 | Length of lateral spike (cm) | 2407 | 1426 | 3833 | 2408 | 2245 | 4653 |
| 10 | Number of spikelets per lateral spike | 3578 | 2301 | 5879 | 3067 | 2702 | 5769 |
| 11 | Number of grains per lateral spike | 3642 | 2293 | 5935 | 2046 | 1605 | 3651 |
| 12 | Grain weight per lateral spike (g) | 3583 | 2215 | 5798 | 569 | 736 | 1305 |

Supplementary Table 4. Image-based traits characterized by large correlations with after-harvest traits.

| Trait no. | Trait name | Trait description | Drought effect cluster | Genetic correlation cluster | Correlation with spike traits cluster | Correlation with TGW cluster | Selected for spike traits | Selected for TGW | No. correlations with spike traits | No. correlations with TGW |
| --- | --- | --- | --- | --- | --- | --- | --- | --- | --- | --- |
| 16 | hull.circularity..geometry.trait.based.on.fluorescence.side.view. | Indicates similarity of the convex hull to a circle, ranges between 0 and 1. A circular object has value 1. | 5 | 7 | 7 | 5 | 1 | 1 | 23 | 4 |
| 39 | area..geometry.trait.based.on.visible.light.side.view...px.2. | Number of foreground pixels. Therefore, projected plant area in pixels. | 6 | 7 | 7 | 5 | 0 | 1 | 14 | 5 |
| 75 | hull.pc2..geometry.trait.based.on.visible.light.side.view...px. | If a line connects the two most far from each other situated plant pixels is drawn, this number indicates the sum of the maximum distances of other plant pixels from the left and right of this line. | 6 | 3 | 7 | 5 | 1 | 1 | 20 | 5 |
| 104 | hsv.h.mean..color.related.trait.based.on.fluorescence.side.view. | Mean - first order texture property (independent of pixel neighbors). Calculated on grayscale image derived from channel Hue (HSV). | 5 | 5 | 3 | 4 | 1 | 1 | 33 | 10 |
| 115 | hsv.v.mean..color.related.trait.based.on.fluorescence.side.view. | Mean - first order texture property (independent of pixel neighbors). Calculated on grayscale image derived from channel Brightness (HSV). | 5 | 7 | 1 | 4 | 0 | 1 | 13 | 5 |
| 124 | intensity.phenol.mean..color.related.trait.based.on.fluorescence.side.view. | A relative indicator of the yellow fluorescence intensity, not taking into account brightness but only the color hue (red = no intensity, yellow = high intensity). Detailed information will be added to the documentation. | 4 | 4 | 1 | 4 | 1 | 0 | 27 | 10 |
| 135 | lab.b.stddev..color.related.trait.based.on.fluorescence.side.view. | The standard deviation of the b values in the L*a*b* colour space of the plant pixels. The lower this value, the more uniform is the plant colour | 5 | 4 | 1 | 4 | 0 | 1 | 15 | 8 |
| 139 | hsv.h.yellow2green..color.related.trait.based.on.visible.light.side.view. | Proportion of yellow colour plant pixels (histogram bin 3) divided by the count of green colour pixels (bins 4 to 7). This value is only valid if the bin count has not been changed from 20, otherwise the involved bins represent different colors. | 3 | 7 | 3 | 1 | 1 | 0 | 40 | 1 |
| 141 | hsv.h.skewness..color.related.trait.based.on.visible.light.side.view. | The 'skewness' of the hue values of the plant pixels. 'skewness' is a statistical term, indicating the tendency of the value distribution to lean to one side of the value range. The documentation will include a more complete description of this trait in the future; see reference literature for full details. | 5 | 6 | 3 | 1 | 1 | 0 | 28 | 1 |
| 143 | hsv.h.mean..color.related.trait.based.on.visible.light.side.view. | Mean - first order texture property (independent of pixel neighbors). Calculated on grayscale image derived from channel Hue (HSV). | 5 | 5 | 6 | 5 | 1 | 0 | 34 | 1 |
| 144 | hsv.h.brown2green..color.related.trait.based.on.visible.light.side.view. | Proportion of brown colour plant pixels (histogram bin 2) divided by the count of green colour pixels (bins 4 to 7). This value is only valid if the bin count has not been changed from 20, otherwise the involved bins represent different colors. | 3 | 5 | 3 | 4 | 1 | 1 | 33 | 3 |
| 150 | lab.a.stddev..color.related.trait.based.on.visible.light.side.view. | The standard deviation of the a-values in the L*a*b* colour space of the plant pixels. The lower this value, the more uniform is the plant colour. | 5 | 5 | 2 | 4 | 1 | 0 | 20 | 0 |
| 154 | hsv.v.skewness..color.related.trait.based.on.visible.light.side.view. | The 'skewness' of the brightness values of the plant pixels. 'skewness' is a statistical term, indicating the tendency of the value distribution to lean to one side of the value range. The documentation will include a more complete description of this trait in the future; see reference literature for full details. | 6 | 7 | 7 | 5 | 1 | 0 | 28 | 2 |
| 157 | rgb.red.mean..color.related.trait.based.on.visible.light.side.view. | Average intensity of the red channel of the plant pixels in the visible light image. | 5 | 7 | 2 | 4 | 1 | 0 | 21 | 2 |
| 166 | rgb.g.std..texture.trait.based.on.fluorescence.side.view. | Standard Deviation - first order texture property (independent of pixel neighbors). Calculated on grayscale image derived from channel Green (RGB). | 4 | 4 | 1 | 4 | 0 | 1 | 16 | 11 |
| 174 | rgb.r.std..texture.trait.based.on.fluorescence.side.view. | Standard Deviation - first order texture property (independent of pixel neighbors). Calculated on grayscale image derived from channel Red (RGB). | 5 | 4 | 1 | 4 | 1 | 0 | 18 | 7 |
| 194 | hull.area..zoom.corrected.geometry.trait.based.on.fluorescence.top.view...mm.2. | Normalized area (in real-world coordinates) of the convex hull, which is the shortest convex line drawing around the plant. | 6 | 6 | 7 | 5 | 1 | 0 | 17 | 1 |
| 256 | hull.compactness.16..geometry.trait.based.on.visible.light.top.view. | borderPixels * borderPixels / filledArea (all of convex hull) | 4 | 7 | 3 | 4 | 1 | 1 | 21 | 3 |
| 261 | leaf.length.sum..geometry.trait.based.on.visible.light.top.view...px. | Skeleton length. | 6 | 7 | 6 | 5 | 1 | 0 | 32 | 0 |
| 269 | hull.pc2..geometry.trait.based.on.visible.light.top.view...px. | If a line connects the two most far from each other situated plant pixels is drawn, this number indicates the sum of the maximum distances of other plant pixels from the left and right of this line. | 6 | 6 | 6 | 5 | 1 | 0 | 26 | 0 |
| 316 | ndvi..color.related.trait.based.on.multi.camera.top.view. | ndvi = (averageNir - averageVisR) / (averageNir + averageVisR)) | 5 | 7 | 6 | 5 | 1 | 0 | 31 | 0 |
| 320 | hsv.v.mean..color.related.trait.based.on.visible.light.top.view. | Mean - first order texture property (independent of pixel neighbors). Calculated on grayscale image derived from channel Brightness (HSV). | 5 | 4 | 3 | 4 | 1 | 0 | 24 | 2 |
| 321 | hsv.s.stddev..color.related.trait.based.on.visible.light.top.view. | The standard deviation of the saturation values of the plant pixels. The lower this value, the more uniform is the saturation of the plant colours. | 5 | 4 | 6 | 3 | 0 | 1 | 16 | 6 |
| 325 | hsv.h.stddev..color.related.trait.based.on.visible.light.top.view. | The standard deviation of the hue values of the plant pixels. The lower this value, the more uniform is the plant colour. | 5 | 7 | 2 | 3 | 0 | 1 | 14 | 3 |
| 330 | lab.b.stddev..color.related.trait.based.on.visible.light.top.view. | The standard deviation of the b values in the L*a*b* colour space of the plant pixels. The lower this value, the more uniform is the plant colour | 5 | 5 | 7 | 3 | 1 | 0 | 19 | 0 |
| 337 | rgb.red.mean..color.related.trait.based.on.visible.light.top.view. | Average intensity of the red channel of the plant pixels in the visible light image. | 5 | 7 | 3 | 4 | 1 | 0 | 30 | 0 |
| 353 | rgb.b.std..texture.trait.based.on.visible.light.top.view. | Standard Deviation - first order texture property (independent of pixel neighbors). Calculated on grayscale image derived from channel Blue (RGB). | 5 | 4 | 6 | 3 | 1 | 0 | 20 | 5 |

Supplementary Table 5. Characteristics of traits selected on the basis of frequent correlation with post-harvest traits – both main spike traits and TGW.

| Trait no. | Trait description | Profile of | | | |
| --- | --- | --- | --- | --- | --- |
|  |  | drought effect | genetic correlation | correlation with main spike traits | correlation with TGW |
| 16 | Indicates similarity of the convex hull to a circle, ranges between 0 and 1. A circular object has value 1 | 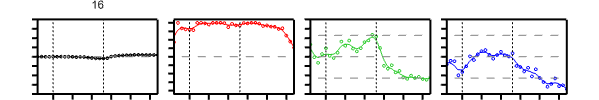 | | | |
| 75 | If a line connects the two most far from each other situated plant pixels is drawn, this number indicates the sum of the maximum distances of other plant pixels from the left and right of this line. | 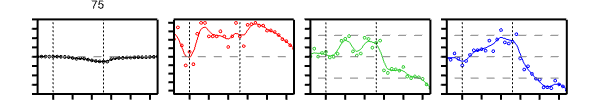 | | | |
| 104 | Mean - first order texture property (independent of pixel neighbors). Calculated on grayscale image derived from channel Hue (HSV). | 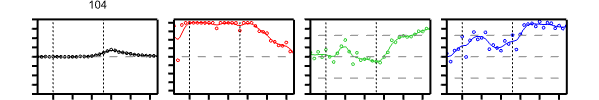 | | | |
| 144 | Proportion of brown colour plant pixels (histogram bin 2) divided by the count of green colour pixels (bins 4 to 7). This value is only valid if the bin count has not been changed from 20, otherwise the involved bins represent different colors. | 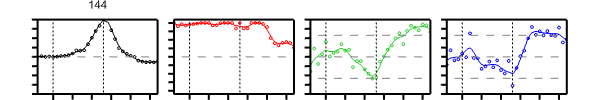 | | | |
| 256 | borderPixels * borderPixels / filledArea (all of convex hull) | 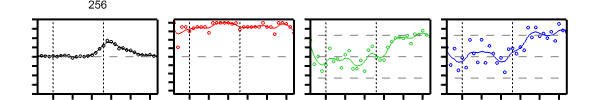 | | | |

| A | B |
| --- | --- |
| 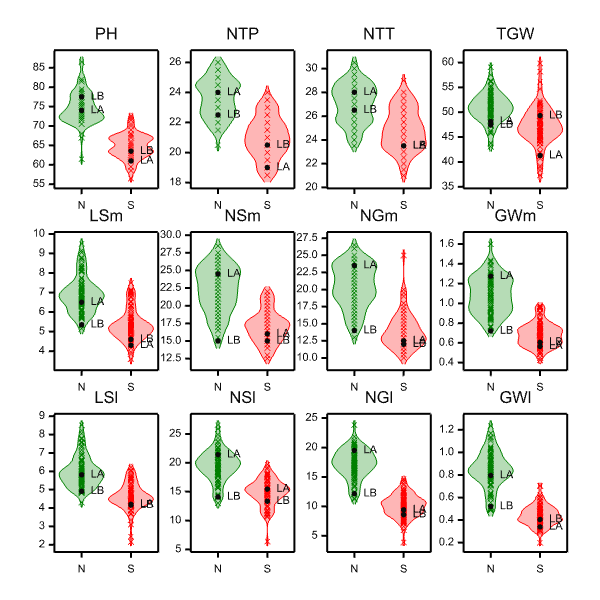 | 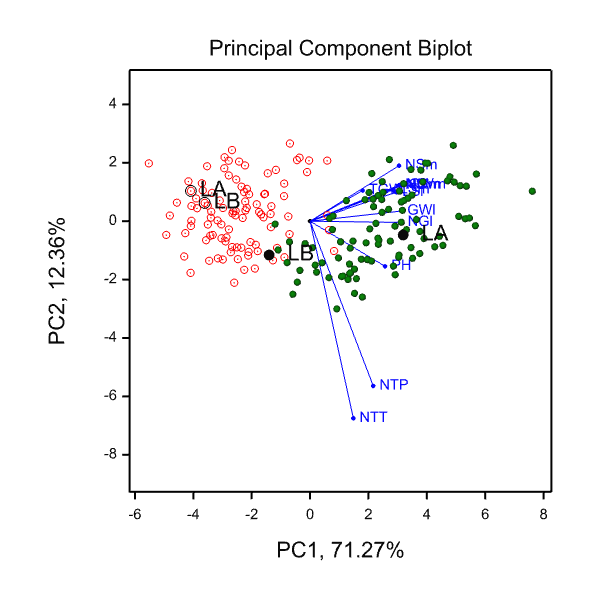 |
| C |  |
| 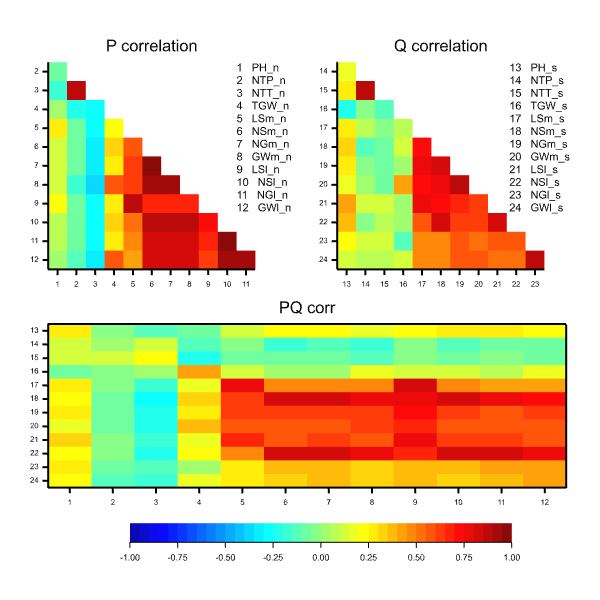 |  |

Supplementary Figure 1.
A. Density plots for traits measured after harvesting (mean values for lines) under optimal (N) and stress (S) conditions. Marked lines LA, LB.
B. Biplot for traits measured after harvesting. Green dots - lines under control conditions, red circles - lines under stress conditions; larger black symbols mark lines LA and LB.
C. Correlations for traits observed after harvest (computed on mean values for lines); 1-12 – traits observed in control conditions (set P), 13-24 – traits observed in drought conditions (set Q).

|  | A |  | B |
| --- | --- | --- | --- |
| WSS (1000) | 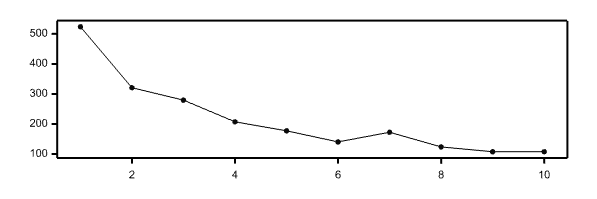 | WSS | 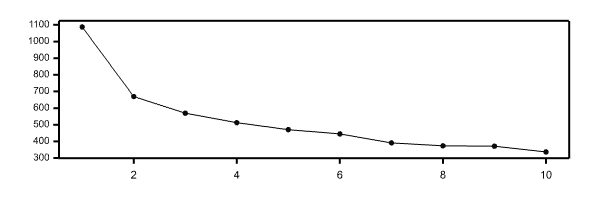 |
|  | Number of groups |  | Number of groups |
|  | C |  | D |
| WSS | 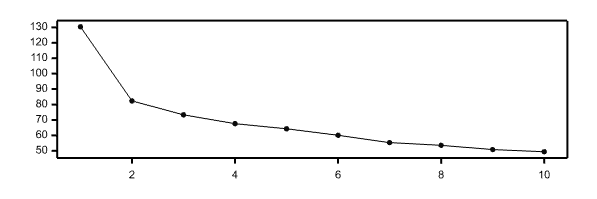 | WSS | 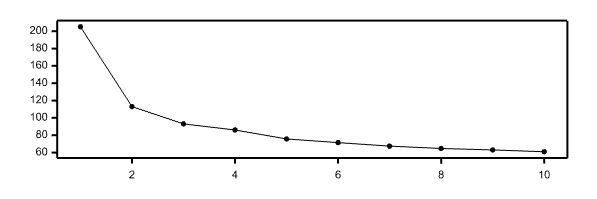 |
|  | Number of groups |  | Number of groups |

Supplementary Figure 2. Within-group sum of squares (WSS) for numbers of clusters from 1 to 10 for clustering:
(A) profiles of mean drought effects for image-derived traits,
(B) profiles of genetic correlation between conditions,
(C) profiles of correlation between relative drought effects (RDE) for image traits and RDE for main spike traits,
(D) profiles of correlation between RDE for image traits and RDE for TGW.

| Yellow to green ratio, VIS, side view, trait 139, cluster (3,7) | Skewness of hue values, VIS, side view, trait 141, cluster (5,6) | 1st order (based on single pixels) texture, std.dev., gray derived from green RGB, FLUOR, side view, trait 166, cluster (4,4) |
| --- | --- | --- |
| 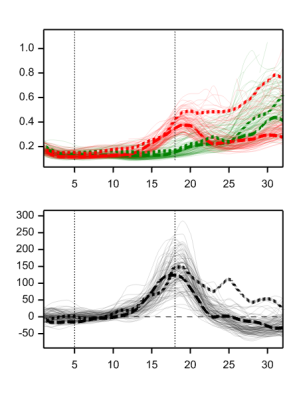  Day | 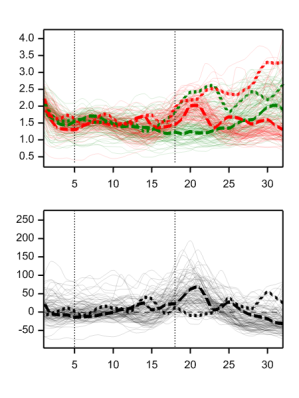  Day | 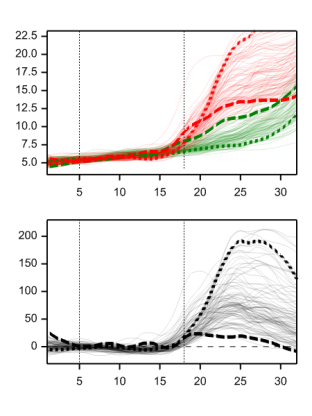  Day |
| Hull compactness, VIS, top view, trait 256, cluster (4,7) | Skeleton length, VIS, top view, trait 261, cluster (6,7) | Std. dev. of hue - color nonuniformity, VIS, top view, trait 325, cluster (5,7) |
| 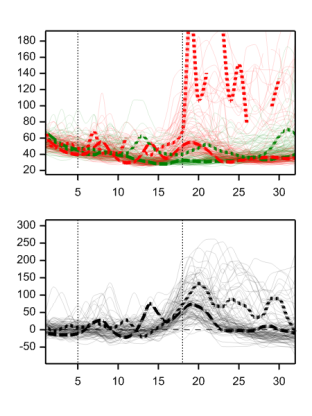  Day | 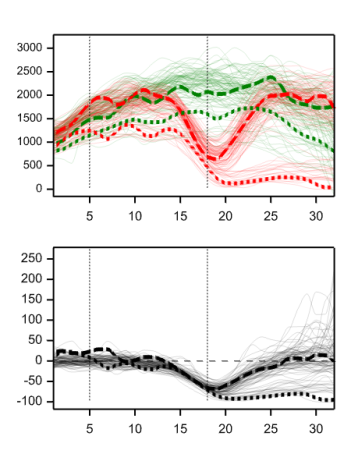  Day | 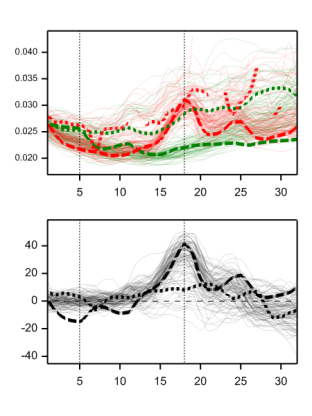  Day |

Supplementary Figure 3. Image-derived traits identified as significant for prediction of main spike traits and TGW. Top rows: Observations for plants grown under normal (green) and stress (red) conditions (mean values over two carriers). Bottom rows: Relative drought effects for all genotypes. Genotype LA – dashed line (high yield in control, big loss in drought), genotype LB - dotted line (low yield under control, little loss in drought).

| A | B |
| --- | --- |
| 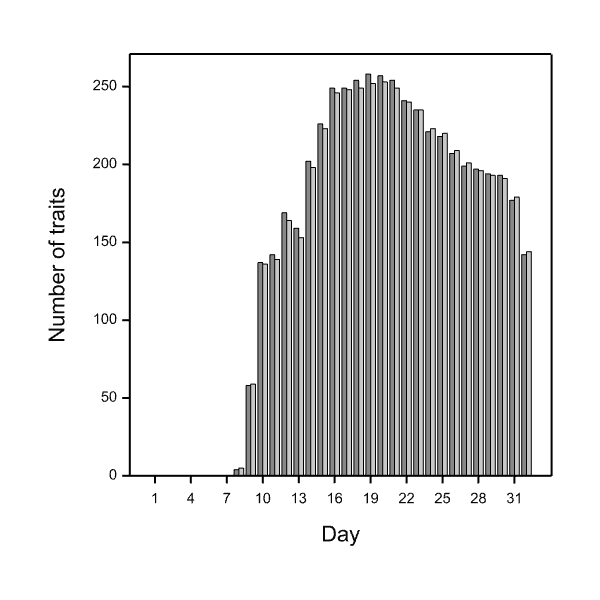 | 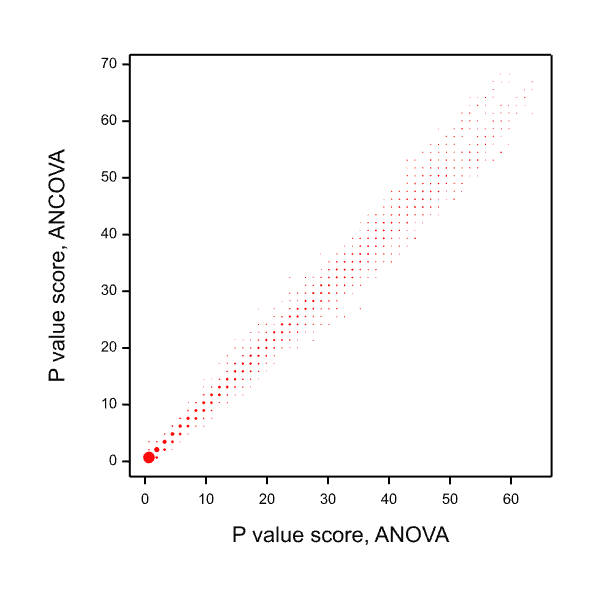 |
| C | D |
| 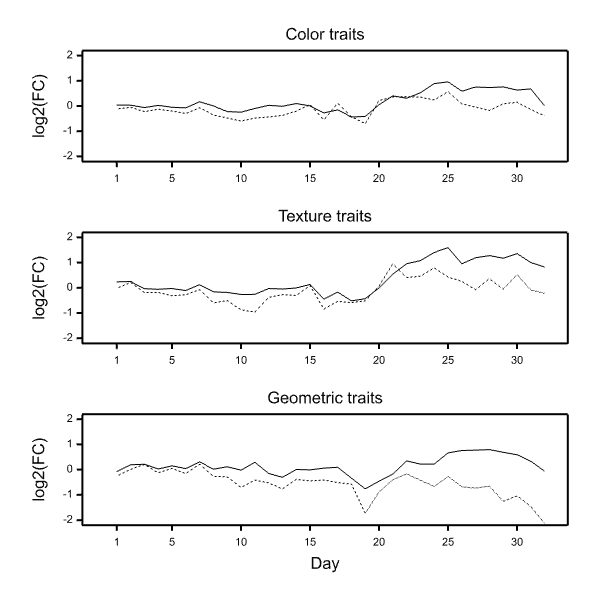 | 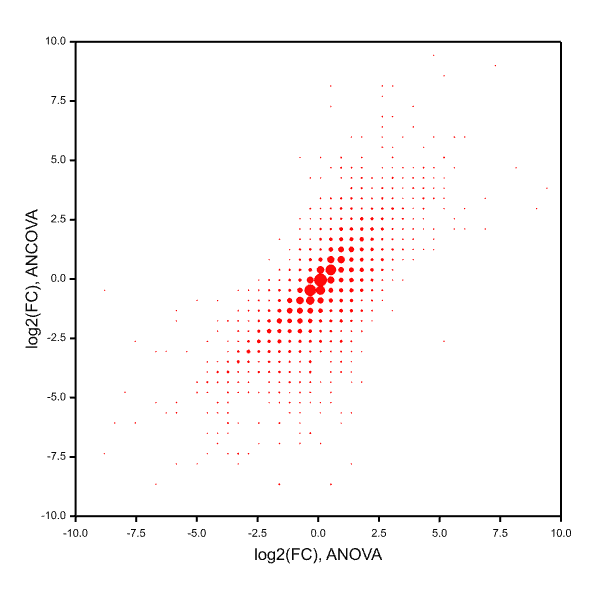 |

Supplementary Figure 4. Comparison of results of statistical analysis based on the linear mixed model applied in Section 4.2 (ANOVA model) and on the model with data on reaching BBCH49 as an additional covariate (ANCOVA model). A. Number of traits with a significant mean drought effect on consecutive days of experiment; dark gray - ANOVA (results as in Figure 2A), light gray - ANCOVA. B. Distribution plot of scores (-log10(P value)) for mean drought effects estimated and tested in the ANOVA and ANCOVA model; included are points representing all traits at all time points. C. Fold change of genetic variance under drought to genetic variance under optimal conditions on consecutive days; continuous line - ANOVA (results as in Figure 2B), dashed line - ANCOVA; the profiles represent mean values in the groups of traits. D. Distribution plot of fold change values (for genetic variance) estimated in ANOVA and ANCOVA model, for all traits at all time points.
